# Low‐Irradiance Antimicrobial Blue Light‐Bathing Therapy for Wound Infection Control

**DOI:** 10.1002/advs.202412493

**Published:** 2025-04-14

**Authors:** Jie Hui, Wonjoon Moon, Pu‐Ting Dong, Carolina dos Anjos, Laisa Negri, Hao Yan, Ying Wang, Joshua Tam, Tianhong Dai, R. Rox Anderson, Jeremy Goverman, Jeffrey A. Gelfand, Seok‐Hyun Yun

**Affiliations:** ^1^ Wellman Center for Photomedicine Massachusetts General Hospital Boston MA 02139 USA; ^2^ Department of Dermatology Harvard Medical School Boston MA 02115 USA; ^3^ Department of Microbiology The ADA Forsyth Institute Boston MA 02142 USA; ^4^ Harvard‐MIT Health Sciences and Technology Cambridge MA 02139 USA; ^5^ Department of Surgery Harvard Medical School Boston MA 02115 USA; ^6^ Wound Center Massachusetts General Hospital Boston MA 02114 USA; ^7^ Division of Infectious Diseases Massachusetts General Hospital Boston MA 02114 USA

**Keywords:** antimicrobial blue light, antimicrobial resistance, medical device, phototherapy, wound infection

## Abstract

The prevalence of antibiotic resistance and tolerance in wound infection management poses a serious and growing health threat, necessitating the exploration of alternative approaches. Antimicrobial blue light therapy offers an appealing, non‐pharmacological solution. However, its practical application has been hindered by the requirement for high irradiance levels (50–200 mW/cm^2^), which particularly raises safety concerns. Here, a light‐bathing strategy is introduced that employs prolonged, continuous exposure to blue light at an irradiance range lower by more than an order of magnitude (5 mW/cm^2^). This method consistently applies bacteriostatic pressure, keeping wound bioburden low, all while minimizing photothermal risks. Leveraging tailor‐made, wearable light‐emitting patches, preclinical trials on rat models of wound infection are conducted, demonstrating its safety and efficacy for suppressing infections induced by methicillin‐resistant *Staphylococcus aureus* (*S. aureus*) and multidrug‐resistant *Pseudomonas aeruginosa* (*P. aeruginosa*). The results pave a new way for the application of blue light therapy in wound care.

## Graphical abstract

Antimicrobial blue light has been shown as an appealing non‐pharmacological antimicrobial alternative. However, its clinical applications have been hindered by high irradiance‐associated photothermal risks. In this work, a low‐irradiance blue light‐bathing concept and a wearable photonic wound patch are established to address this challenge in terms of its safety, effectiveness, and potential practical use in wound care.

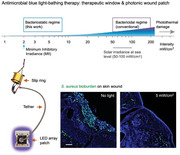

## 1 Introduction

Wound infection is a major worldwide healthcare burden.^[^ ^1^, ^2^, ^3^ ^]^ The requirement for orchestrated healing factors and the ease of exposure to microbial contaminants render wounds, especially chronic wounds, susceptible to infection.^[^ ^3^, ^4^, ^5^, ^6^ ^]^ If the infection is not properly managed, it can lead to delayed healing and serious or even life‐threatening complications.^[^ ^3^, ^7^ ^]^ Both systemic antibiotics and topical antimicrobials are crucial for treating invasive infections. However, they encounter significant challenges in wound infection management, particularly associated with antimicrobial resistance, biofilm formation, and adverse effects. It is estimated that over 70% of bacteria responsible for wound infections are resistant to at least one common antibiotic.^[^ ^8^, ^9^ ^]^ This situation is further complicated by the formation of bacterial biofilms, which can display remarkably high antibiotic tolerance compared to their planktonic counterparts.^[^ ^10^, ^11^, ^12^ ^]^ Current clinical guidelines advise the careful use of topical antimicrobials and systemic antibiotics and recommend limiting antiseptics to short‐term use to avoid toxicity and hypersensitivity reactions.^[^ ^13^, ^14^, ^15^, ^16^ ^]^ These challenges highlight the urgent need for the development of innovative antimicrobial strategies.

Current antibiotic discovery has encountered significant obstacles as no new class of antibiotics has been introduced into the clinic in the last 4 decades.^[^ ^17^, ^18^ ^]^ At the same time, antimicrobial resistance has become a paramount public health crisis,^[^ ^19^, ^20^, ^21^, ^22^ ^]^ contributing to 1.27 million deaths globally in 2019 and projected to cause 10 million annually by 2050.^[^ ^2^ ^]^ The dwindling antibiotic discovery pipeline, combined with the rising antimicrobial resistance, has already propelled us into a post‐antibiotic era. In response, the scientific community has investigated various alternatives, particularly those with novel antimicrobial mechanisms, minimized selective pressure, or reduced off‐target issues. Techniques encompassing anti‐virulence therapies, antimicrobial peptides, phage therapy, and antibodies have shown promise in early research,^[^ ^18^, ^23^, ^24^ ^]^ yet their clinical adoption remains elusive. Among these non‐antibiotic approaches, antimicrobial blue light therapy has received significant attention. This approach targets endogenous chromophores in microbes, particularly porphyrin derivatives, and produces bacteria‐specific cytotoxic effects upon blue light illumination (400–470 nm). Owing to its non‐pharmacological nature and broad‐spectrum efficacy, blue light therapy is considered a promising avenue^[^ ^25^, ^26^, ^27^, ^28^, ^29^, ^30^, ^31^, ^32^, ^33^, ^34^ ^]^ for localized antimicrobial use, especially in wound care. Extensive laboratory and animal studies indicate its antimicrobial potency against many common wound pathogens.^[^ ^32^, ^33^, ^34^, ^35^, ^36^, ^37^, ^38^, ^39^, ^40^, ^41^, ^42^ ^]^ Nevertheless, its clinical translation has faced substantial obstacles due to its limited bactericidal effectiveness in vivo and high photothermal risks resulting from high‐irradiance/intensity illumination.

Current blue light therapy protocols aim to rapidly eliminate bacteria using single or multiple doses, each lasting from 10 min to 1 h, necessitating high optical intensities exceeding tens of milliwatts (mW) per cm^2^. Intensities between 50–100 mW/cm^2^ have shown potent bactericidal efficacy in vitro in a dose‐dependent manner.^[^ ^29^, ^33^, ^35^, ^37^, ^39^, ^40^ ^]^ However, when advanced to in vivo testing on animal models, even 200 mW/cm^2^—considered the maximum permissible exposure level given by the American National Standard Institute^[^ ^43^ ^]^—at 300 J cm^−2^ levels only results in moderate (< 2‐log_10_) reductions.^[^ ^32^, ^42^ ^]^ These high‐intensity levels pose a serious risk of photothermal damage, which becomes more prominent under repeated dosing to prevent bacterial relapse.^[^ ^32^, ^42^, ^44^ ^]^ Moreover, the demand for high irradiance places substantial practical constraints on light sources, especially for large wounds which require high total power and proper thermal management for large‐area illumination. This requirement can present an even bigger challenge for wearable device development.^[^ ^45^, ^46^ ^]^

Here, we introduce a promising solution to this bottleneck, challenging traditional approaches. We suggest that antimicrobial blue light therapy may not be best suited for quickly eliminating bacteria in infected wounds. Instead, its most effective use could be as a prolonged intervention during wound healing, applying continuous antimicrobial pressure on the wound bed to maintain its bacterial load below a clinically acceptable level. This revised strategy, termed “light‐bathing”, involves continuous or quasi‐continuous exposure to blue light in a low‐irradiance paradigm. In this paper, we present highly encouraging results from our in vitro and in vivo studies of this light‐bathing approach, utilizing custom‐built, wearable light‐emitting patches on rat models of wound infection. We discovered that prolonged exposure to 410‐nm light at just 5 mW/cm^2^ can effectively prevent bacterial growth and keep the in vivo wound bioburden low for both Gram‐positive *S. aureus* and Gram‐negative *P. aeruginosa*, two of the most common antibiotic‐resistant wound pathogens, over the entire 2‐day treatment course. Its safety and effectiveness were rigorously confirmed through various methods, including clinical wound signs, immunohistochemical analysis, bioluminescence imaging (BLI), fluorescence in situ hybridization (FISH) imaging, and endpoint colony‐forming unit (CFU) enumeration from wound biopsies. Our findings highlight the potential of blue light‐bathing as a new antimicrobial strategy in wound care.

## 2 Results

### 2.1 Minimum Irradiance Levels for Bacterial Growth Inhibition

**Figure 1:**
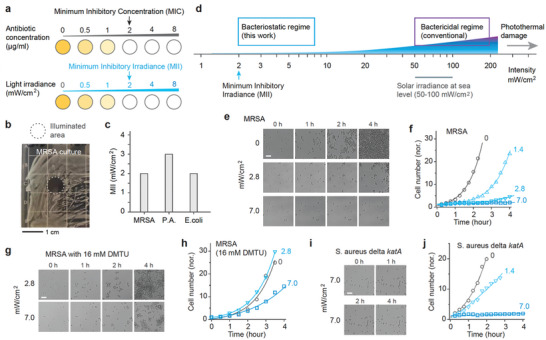
MII concept and the proposed therapeutic window for bacteriostatic blue light therapy. a) Illustration of MII, defined as the lowest irradiance that enables complete bacterial growth inhibition under continuous 410 nm illumination, akin to the concept of MIC for antibiotics. b) Image of an MRSA‐streaked agar plate following overnight continuous exposure to 410 nm blue light at 2 mW/cm^2^, highlighting the treated area (dotted circle) through an 8‐mm diameter aperture. c) Measured MII levels for three prevalent wound pathogens: MRSA (USA300), *P. aeruginosa* (ATCC 27853), and *E. coli* (ATCC 25922). d) Diagram illustrating the proposed bacteriostatic window which employs significantly lower light intensities compared to traditional high‐irradiance bactericidal approaches, which are comparable to solar irradiance at sea level under clear skies. Noteworthy, “bacteriostatic” and “bactericidal” are used to describe the two irradiance windows, particularly to highlight their different clinical end goals. e) Time‐lapse optical imaging of MRSA USA300 cells under continuous blue light exposure at varying irradiances. All cell groups underwent similar preparation and analysis. f) Growth curves for MRSA, derived from the sequential images in (e). g) Time‐lapse imagery of MRSA USA300 cells treated with 16 mMm DMTU to deplete ROS. h) Growth curves for the ROS‐depleted MRSA. i) Time‐lapse imagery of catalase‐deficient mutant *S. aureus* Δ*katA*. j) Growth curves for *S. aureus* Δ*katA*. Each growth curve was analyzed using an exponential function to estimate cell doubling time. The experiments shown in panels (b–j) were performed in duplicate to ensure reproducibility. Scale bars represent 20 µm.

In conventional antimicrobial discovery and susceptibility testing, the “Minimum Inhibitory Concentration” (MIC) is a widely used benchmark to characterize the lowest concentration of an antimicrobial agent that visibly inhibits the growth of a microorganism on agar plates or in broth media^[^ ^47^ ^]^ (refer to **Figure** **1**a). Typically, an antibiotic's effectiveness is gauged by maintaining a concentration at or above the MIC, with dosages adjusted as necessary. Drawing inspiration from this established metric, we introduced the concept of “Minimum Inhibitory Irradiance” (MII), which we define as the minimum irradiance of blue light required under continuous long‐term illumination (e.g., overnight or longer) to enable complete growth inhibition of a microorganism within an in vitro growth environment. To quantify MII, we conducted an in vitro assay involving the incubation of bacterial cultures on agar plates followed by concurrent overnight incubation at 37 °C and continuous 410 nm light illumination at varying irradiances as illustrated in Figure 1a. The growth of methicillin‐resistant *S. aureus* (MRSA USA300), a prevalent wound pathogen noted for its tolerance to 410 nm light,^[^ ^29^ ^]^ was fully inhibited after a 24‐h exposure to light irradiance of 2 mW/cm^2^ (Figure 1b), while irradiances below 1 mW/cm^2^ induced visibly slower bacterial growth. Post‐treatment, an additional 24‐h culture in darkness showed no relapse within the illuminated zones (Figure S1, Supporting Information). Therefore, the MII for MRSA (USA300) was determined to be 2 mW/cm^2^. By the same method, the MIIs for *P. aeruginosa* (ATCC 27853) and *E. coli* (ATCC 25922) were determined to be 3 and 2 mW/cm^2^, respectively (Figure 1c). These MII values are more than an order of magnitude lower than the typical irradiances employed in traditional blue light therapy (Figure 1d). At such low irradiances, light‐bathing—continuous illumination over extended periods—can be employed as a new regimen to ensure a sustained and sufficient antimicrobial pressure.

### 2.2 Bacteriostatic Effects of Low‐Irradiance Light‐Bathing on In Vitro Culture

To delve deeper into the antimicrobial effects of light‐bathing, we observed the growth dynamics of various bacterial strains cultured in brain heart infusion (BHI) media under continuous 410 nm illumination. In the absence of light, MRSA cells exhibited rapid proliferation (Movie S1, Supporting Information), with a cell doubling time of approximately 29 min as deduced from the growth curve fitting (Figure 1e,f). Continuous exposure to light at irradiances of 1.4 and 2.8 mW/cm^2^ resulted in noticeable retardation of cell growth, extending cell doubling time to 52 and 128 min, respectively. At an irradiance of 7 mW/cm^2^, we observed a complete halt in cell proliferation. This level of irradiance surpasses the MII of 2 mW/cm^2^ identified for streaked agar plates, a variance attributed to both the rich nutrients and optical absorption of the media (Figure S2, Supporting Information).

Similar to conventional high‐irradiance blue light therapy,^[^ ^27^, ^29^, ^32^ ^]^ the antimicrobial action at lower irradiances was presumed to stem from the generation of reactive oxygen species (ROS). To validate this hypothesis, we introduced N,N'‐dimethylthiourea (DMTU), a ROS scavenger, into the culture medium at a concentration of 16 mM and subsequently monitored the growth under varying light irradiances (Figure 1g and Movie S2, Supporting Information). In the presence of DMTU, the difference in bacterial growth between the unilluminated control and the 2.8 mW/cm^2^ illuminated group was negligible. Notably, at 7 mW/cm^2^, bacterial proliferation was no longer inhibited effectively (Figure 1h). This corroborates the ROS‐mediated antimicrobial mechanism of light‐bathing.

Further exploration involved subjecting catalase‐deficient mutant *S. aureus* (Δ*katA*) to light‐bathing. Prior research has identified catalase as a critical molecular target for blue light, implicated in the disruption of H_2_O_2_ detoxification processes.^[^ ^31^, ^32^ ^]^ Contrary to our expectation, light‐bathing exhibited comparable efficacy in both the catalase‐deficient mutant and MRSA strains (Figure 1i,j; Movie S3, Supporting Information). This observation suggests that other bacterial chromophores or mechanisms, particularly staphyloxanthin^[^ ^30^, ^34^ ^]^ and porphyrins,^[^ ^29^, ^48^ ^]^ might also be actively involved in the antimicrobial effect of light‐bathing. While this aspect merits further investigation, the multi‐molecular‐target mechanism could implicate a reduced likelihood of resistance development.

### 2.3 Animal Model and Light‐Delivering Wound Patch for In Vivo Testing

**Figure 2:**
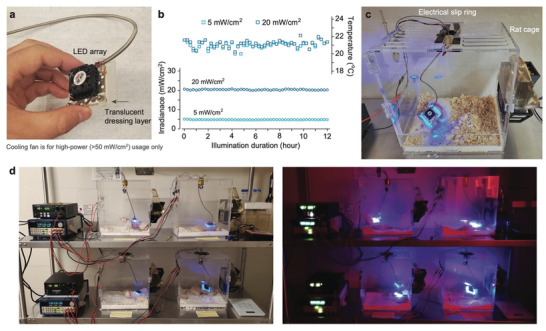
Design of antimicrobial photonic wound patch and prototype of a tethered, wearable device for in vivo rat studies. a) Image of the wearable light source featuring an LED array, cooling components for the LED, and a custom 3D‐printed mounting frame. b) Graph showing the stability of the LED's output irradiance and temperature when operated without active cooling at 5 and 20 mW/cm^2^. c) Photograph of the tethered, wearable device prototype fitted on a freely moving rat, illustrating the device in use with active blue light illumination. d) Overview of four complete sets of the device‐cage system, including peripheral components, designed to enhance study throughput and validate reproducibility. Images capture rats under active light treatment, depicted with and without the presence of ambient lighting.

To extend our examination of the light‐bathing strategy to live animals, a device capable of delivering light continuously to target sites is needed. We devised wearable patches equipped with blue light‐emitting diodes (LED) and engineered a tethered prototype optimized for use with freely moving rat models (**Figure** **2**a and Movie S4, Supporting Information). This device comprised a 10×10 LED array, covering a 22×22 mm^2^ area, emitting light at a spectrum of 409±5.5 nm, with tunable output intensity (Figure S3, Supporting Information). The quality of each LED array used in the study was assessed by its qualitative brightness at single LED level (Figure S3, Supporting Information) followed by quantitative evaluation of its irradiance uniformity across rat skin wound‐equivalent area (Figure S4, Supporting Information). Their high‐density array packing and divergent emission angle (120–140°) yielded a highly overlapping illumination field, warranting a relatively uniform illumination across the entire 15×15 mm^2^ area (with a coefficient of variation of approximately 8%) in a depth range of 2–5 mm from its emitting facet (Figures S4 and S5, Supporting Information). To mitigate the potential overheating of the device, we fitted it with a heat sink and a miniature cooling fan, enabling an output of up to 200 mW/cm^2^ during biosafety assessment. However, we found that active cooling was not necessary at therapeutic irradiances below 20 mW/cm^2^, where without cooling, the LED's output was stable, and device heating was negligible (Figure 2b).

For animal trials, we selected the dorsal region of rats as the optimal site for both device placement and subsequent abrasion wound creation. Following the induction of wounds, we applied a standard transparent film drape to seal the wound to prevent external contamination before mounting the LED headpiece. The assembled wound patch was mounted onto intact dorsal skin, maintaining a distance of approximately 5 mm between the LED emitting facet and tissue (Figure S6, Supporting Information). During later in vivo rat studies, we monitored the thermal condition of the LED arrays and found no heating issues throughout the treatment course. To accommodate the unique requirements of our study, rat cages were customized to securely house the tethered wound patch device (Figure 2c). The design and refinement of the cage setup were achieved through iterative testing and optimization. In particular, a spring‐loaded tether and an electrical slip ring were incorporated to reduce restrictions on rat mobility and to prevent cable entanglement or chewing. We constructed four identical device‐cage setups to enable parallel experimentation on four individual subjects for follow‐up in vivo evaluation of phototoxicity and antimicrobial efficacy (Figure 2d and Table S1, Supporting Information).

### 2.4 Phototoxicity Evaluation on Healthy and Wounded Skin

**Figure 3:**
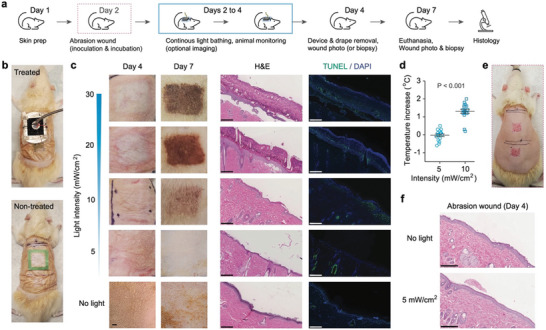
Evaluation of phototoxicity on in vivo rats. a) Diagram detailing the phototoxicity study protocol conducted on healthy or wounded rat dorsal skin. b) Comparative photographs depicting a rat equipped with the light‐delivery device undergoing treatment (top) and a control rat not subjected to light exposure (bottom). c) Bright‐field in vivo images of the healthy skin regions were captured on Days 4 and 7 after 2‐day light treatment at different irradiances, accompanied by corresponding H&E‐stained and TUNEL‐stained histology images from biopsied samples collected on Day 7. Three technical replicates per biological sample and five biological replicates for both the untreated and 5 mW/cm^2^‐treated groups were applied. d) Graph illustrating the temperature increase measured at the dorsal skin surface during continuous illumination. e) Images showing a rat with two freshly created abrasion wounds. f) H&E‐stained histological sections of dorsal abrasion wounds, comparing the untreated versus 2‐day light‐treated at 5 mW/cm^2^. Three technical replicates per biological sample and three biological replicates were applied. Scale bars indicate 250 µm.

While MII sets the lower bound of the therapeutic window depicted in Figure 1d, the risk of phototoxicity determines its upper bound. Our initial investigation focused on assessing the phototoxic effects of prolonged light exposure on healthy skin to establish the Maximum Permissible Irradiance (MPI), prioritizing photothermal safety. The schematic of our study protocol is presented in **Figure** **3**a. After preparatory steps of hair removal and skin cleansing, we equipped the rat's dorsal skin with a light‐transmitting (92%) drape film and an LED headpiece (Figure 3b). We employed an irradiance de‐escalation method. In our initial short‐duration (6 h) toxicity tests, the animals were found to endure an irradiance of 30 mW/cm^2^ without showing significant signs of discomfort, edema, and erythema. Consequently, this irradiance level was selected as the benchmark for the following study with further extended exposure. The rats underwent a continuous 2‐day light exposure at varying irradiances, after which we monitored the skin's response over the next three days, a duration deemed adequate to observe any delayed phototoxic effects.^[^ ^49^ ^]^ This was followed by taking skin biopsies for histopathological examination. This 2‐day exposure period matches the typical interval for changing wound dressings in clinical settings.

Post‐treatment, we observed no notable erythema or edema across all subjects, in line with Draize's criteria used for the toxicity test (Figure 3c). However, animals exposed to higher irradiances of 20 and 30 mW/cm^2^ exhibited marked skin discoloration, indicative of phototoxicity affecting the epidermis. Three days after treatment, these discolored regions progressed to darkened, necrotic tissue layers (Figure 3c), with this effect being less pronounced at 10 mW/cm^2^ and virtually absent at 5 mW/cm^2^. Further microscopic analysis through H&E and TUNEL staining elucidated the presence of 200–300 µm‐thick necrotic layers and apoptotic cells at higher irradiance levels (Figure 3c). Remarkably, samples exposed to 5 mW/cm^2^ showed no evidence of tissue necrosis or cellular apoptosis. Temperature measurements taken directly beneath the LED patch revealed no significant increase at 5 mW/cm^2^, whereas a modest rise of 1.3 °C was noted at 10 mW/cm^2^ (Figure 3d). Based on these results, we set the MPI range for rat skin between 5 and 10 mW/cm^2^.

Further evaluations were conducted on abrasion‐induced wounds on the rat's dorsal skin (Figure 3e), mimicking superficial injuries by disrupting the stratum corneum and epidermis layers (Figure S7, Supporting Information). In the absence of light exposure, these non‐contaminated wounds naturally healed within 48 h. Histological examination confirmed similar regeneration of the stratum corneum and epidermis in areas treated with light (Figure 3f), indicating that 2‐day light‐bathing at 5 mW/cm^2^ does not hinder the wound healing process. It is important to note that this irradiance level is 40 times lower than the maximum permissible exposure of 200 mW/cm^2^ set by the American National Standard Institute, which has been frequently cited to justify higher irradiances in previous preclinical studies of blue light therapy. Our findings urge caution regarding the safety of using a few tens of milliwatts per cm^2^ in blue wavelengths.

### 2.5 Light‐Bathing Therapy on MRSA‐Infected Wound

To assess the bacteriostatic efficacy of light‐bathing, we integrated contamination and infection phases into our abrasion wound model. We inoculated the wound surfaces, each measuring approximately 15 mm×15 mm, with a high load (approximately 10^8^ CFUs) of MRSA USA300 immediately following the abrasion procedure, covered them with a drape film, and then allowed a 3‐h period for bacterial colonization and infection. Drawing on our MII and MPI values, we selected an irradiance of 5 mW/cm^2^ for all further evaluations. To observe the infection dynamics, we first employed a bioluminescent MRSA strain (MRSA USA300 *lux* ^[^ ^44^ ^]^) and performed time‐lapse BLI^[^ ^50^ ^]^ to monitor the bacterial load over a 44‐h period, encompassing 41 h of light exposure. Additionally, we infected another set of rats with non‐luminescent antibiotic‐resistant clinical isolate (MRSA USA300) following the same protocol and then conducted punch biopsies on wounds^[^ ^51^ ^]^ for comprehensive bioburden and wound condition analyses.

**Figure 4:**
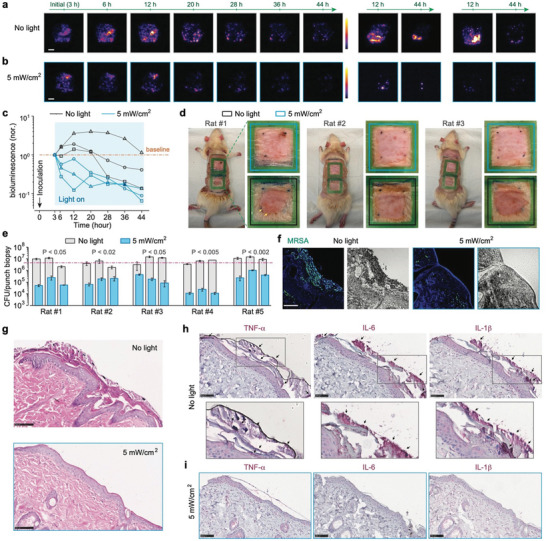
Evaluation of effectiveness on in vivo rat models of MRSA wound infection. a,b) Time‐lapse BLI showed the progression of MRSA USA300 *lux* infection in rat dorsal abrasion wounds, comparing untreated (a) to those receiving 2‐day light therapy at 5 mW/cm^2^ (b). Three sets of images were acquired from three independent biological replicates for each group. c) Plots of relative integrated bioluminescence intensity of each wound, which were normalized to their initial values at 3 h, illustrating the dynamic bacterial load over time. d) Endpoint visual assessment of wound appearance. Yellow arrows indicate infection‐induced pus. e) Endpoint CFU enumeration of bacterial load within punch biopsy samples taken from each wound. The pink dashed line indicates the initial inoculation load. Three technical replicates per biological sample and five biological replicates were applied. *P* values were calculated based on punch biopsy‐based CFUs between the untreated and 5 mW/cm^2^‐treated wounds on the same individual rat. f) Endpoint *S. aureus*‐specific PNA‐FISH imaging provides detailed visualization of bacterial presence in punch biopsy samples from each group. Three technical replicates per biological sample and three biological replicates were applied. g) Endpoint H&E‐stained histological sections highlight tissue morphology. h,i) Endpoint immunohistochemical staining of several key cytokines in samples from the untreated group (h) versus the light‐treated group (i). Arrows indicate the extensive expression of corresponding cytokine markers in the untreated group. Three technical replicates per biological sample and three biological replicates were applied for (g–i). Scale bars, 5 mm for (a) and 100 µm in (f–i).

The BLI signal, proportional to bacterial load with a detection limit of 1.8×10^5^ CFUs/cm^2^ for MRSA USA300 *lux*, indicated that the initial high inoculation load resulted in a robust BLI signal across the infected area (Figure S8–S10, Supporting Information). In the control group, the bacterial load peaked around 12 h post‐inoculation, then gradually declined, likely due to natural host defense mechanisms, although scattered hot spots of activity persisted at 44 h (**Figures** **4**a and S11, Supporting Information). Notably, one control animal exhibited a pus‐like area at 12 h and an ongoing active infection at the 44‐h mark (middle in Figure 4a). By contrast, the bacterial load in the light‐treated group generally started to decrease almost immediately with light treatment applied and continued over time, culminating in nearly complete eradication by the 44‐h endpoint (Figure 4b and Figure S11, Supporting Information). As apparent in the dynamic curves of bacterial load quantified via BLI (Figure 4c), these treated wounds showed significantly lower bioburden levels across all time points, especially notable when compared to the first 20 h of active infection in the control group. Visual assessment further confirmed the cleaner and healthier appearance of treated wounds compared to the inflamed and pus‐producing control wounds (Figure 4d). Endpoint CFU bioburden analysis on wounds infected with non‐luminescent MRSA showcased a substantial reduction in bacterial load in the treated groups, a 2‐log_10_ reduction compared to both the control groups and initial inoculation level (Figure 4e and Figure S12, Supporting Information). FISH imaging using *S. aureus*‐specific peptide nucleic acid probes (PNA) highlighted a drastically decreased bacterial presence, primarily confined to the surface of the treated wounds (Figures 4f and S13, Supporting Information).

Morphological changes and inflammatory response evaluated through H&E and immunohistochemical staining for TNF‐α, IL‐6, and IL‐1β cytokines in the biopsied samples further delineated the therapeutic effect. Control wounds exhibited typical signs of infection‐induced inflammation, including necrotic tissue discharge and epidermal proliferation (Figure 4g). In stark contrast, treated wounds displayed no such pathology, appearing virtually identical to normal, healthy skin. The absence of pro‐inflammatory cytokines in treated samples underscores the therapy's ability to not only control MRSA infection but also facilitate the healing process by mitigating inflammation (Figures 4h,i and S14, Supporting Information). Through these analyses, light‐bathing therapy has demonstrated profound success in managing MRSA‐infected wounds.

### 2.6 Light‐Bathing Therapy on *P. aeruginosa*‐Infected Wound

**Figure 5:**
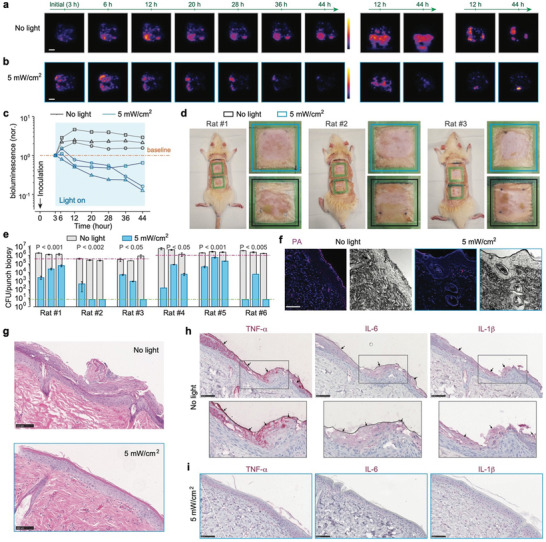
Evaluation of effectiveness on in vivo rat models of *P. aeruginosa* wound infection. a,b) Time‐lapse BLI tracked the progression of *P. aeruginosa* infection in rat dorsal abrasion wounds, showcasing untreated wounds (a) against those subjected to 2‐day light therapy at 5 mW/cm^2^ (b). Three sets of images were acquired from three independent biological replicates for each group. c) Plots of relative integrated bioluminescence intensity of each wound, which were normalized to their initial values at 3 h, illustrating changes in bacterial load over time. d) Endpoint visual assessment of wound appearance. Extensive wound exudates were observed in the untreated wounds. e) Endpoint CFU enumeration of bacterial load within punch biopsies randomly sampled from each wound. The pink and green dashed lines indicate the initial inoculation load and the detection limit, respectively. Three technical replicates per biological sample and six biological replicates were applied. *P* values were calculated based on punch biopsy‐based CFUs between the untreated and 5 mW/cm^2^‐treated wounds on the same individual rat. f) Endpoint *P. aeruginosa*‐specific PNA‐FISH imaging for detailed visualization of bacterial distribution in punch biopsy samples from each group. Three technical replicates per biological sample and three biological replicates were applied. g) Endpoint H&E‐stained histological sections. h,i) Endpoint immunohistochemistry results of key cytokine markers in samples from the untreated group (h) compared to the light‐treated group (i). Arrows indicate the extensive expression of corresponding cytokine markers in the untreated group. Three technical replicates per biological sample and three biological replicates were applied for (g–i). Scale bars indicate 5 mm in (a) and 100 µm for (f–i).

Turning our attention to *P. aeruginosa*, particularly known for its virulence and multidrug resistance, we utilized two different strains, a bioluminescent strain (PAO1 *lux* ^[^ ^28^, ^52^ ^]^) for monitoring time‐lapse infection and a clinical strain (CDC AR Bank #0231) for endpoint analyses, and conducted similar antimicrobial effectiveness evaluation. The *lux* expression level of PAO1 *lux* was found to be more than 200 times higher than that of MRSA USA300 *lux*, offering a minimum detection sensitivity of 7.4×10^2^ CFUs/cm^2^. In the untreated wounds (**Figures** **5**a and S15, Supporting Information), the infection, indicated by the measured bioburden, escalated over time, peaking at 12 h and subsequently leading to the formation of extensive wound exudate (pus), indicating an aggressive infection. Remarkably, the application of light‐bathing at 5 mW/cm^2^ not only curtailed this escalation but progressively reduced the bacterial load over time, as evidenced by the apparently diminishing BLI signal (Figures 5b and S15, Supporting Information), without any noticeable pus formation. This contrast highlights the antimicrobial effectiveness of our light therapy by significantly suppressing the bacterial population below the initial inoculation levels throughout the treatment duration (Figure 5c). Visual inspections further differentiated the outcomes between the treated and untreated groups. The untreated wounds were marred by greenish, spreading exudate visible through the transparent film drape, whereas the treated wounds maintained a much cleaner and healthier appearance (Figure 5d).

Following the same protocol, we used a multidrug‐resistant *P. aeruginosa* clinical isolate (CDC AR Bank #0231) and performed similar bioburden and wound condition analyses. Quantitative analysis through CFU enumeration of wound biopsies revealed a significant reduction, 2–3‐log_10_ CFUs in the treated group, on average, compared to the untreated control (Figures 5e and S16, Supporting Information), with several samples achieving “sterilization” levels with CFUs diminished beyond the detection limit. This result was corroborated by *P. aeruginosa*‐specific PNA‐FISH imaging, which showed a dramatic bacterial reduction, particularly in the superficial epidermal layers of the treated wounds, in contrast to the dense bacterial presence in the untreated wounds (Figures 5f and S13, Supporting Information). Histological evaluations provided further insights into the therapeutic impact of the light‐bathing approach. H&E staining of the untreated wounds displayed thick, infected lesions across the wound surfaces, whereas these treated wounds exhibited normal skin morphology, free from infection‐induced disruptions (Figure 5g). The absence of inflammatory markers such as TNF‐α, IL‐6, and IL‐1β in the treated wounds (Figure 5i), as opposed to their extensive expression in the untreated wounds (Figure 5h), confirmed the dual antimicrobial and anti‐inflammatory efficacy of light‐bathing therapy in managing *P. aeruginosa*‐infected wounds.

## 3 Discussion

Our research represents substantial progress in antimicrobial blue light therapy by introducing a novel bathing regimen that diverges from the traditional protocols which utilize high irradiance levels (50–200 mW/cm^2^) and short treatment durations (10–60 min).^[^ ^29^, ^32^, ^33^, ^35^, ^37^, ^39^, ^40^, ^42^ ^]^ The identification of a new therapeutic window for blue light therapy and the implementation of a wound patch device on freely moving animals are crucial, highlighting their biosafety, effectiveness, and feasibility for clinical use. Contrary to conventional high‐irradiance methods aiming at rapid and complete bacterial eradication, our approach sustains the necessary antimicrobial pressure to maintain bacterial loads below clinically acceptable levels (e.g., < 10^5^ CFUs/g) throughout the treatment. Although photobiomodulation therapy utilizing low‐level irradiances has been clinically established^[^ ^53^, ^54^, ^55^ ^]^ and it could coexist in blue light therapy, its light‐induced cellular stimulation is entirely different from blue light‐mediated antimicrobial effects. As a result, the two modalities exhibit significant differences in their illumination wavelengths, irradiance window, treatment regimen, and device implementation or requirements, making them non‐transferable to each other.

With the identification of key molecular targets, particularly porphyrins,^[^ ^29^, ^42^, ^48^ ^]^ catalase,^[^ ^31^, ^32^ ^]^ and staphyloxanthin,^[^ ^30^, ^34^ ^]^ and their associated antimicrobial mechanisms, the antimicrobial blue light direction has recently experienced a fresh wave of development and clinical translation. When compared to many antimicrobial compounds, this light‐based antimicrobial approach has a lower propensity for resistance development, benefiting from its rich antimicrobial mechanisms. Nevertheless, a more thorough examination of its resistance or tolerance development shall be warranted.^[^ ^56^ ^]^ Additionally, as the expression levels of these endogenous molecular targets vary from strain to strain and from species to species, their susceptibility to blue light therapy could also vary.^[^ ^48^ ^]^ This work focused on some representative strains of Gram‐positive *S. aureus* and Gram‐negative *P. aeruginosa* in vitro and in vivo, which is deemed sufficient to demonstrate the proof‐of‐concept of our light‐bathing therapy and wearable wound patch approaches. For the follow‐up susceptibility studies, we intend to examine more bacterial species and strains.

A limitation of our current study is its focus on acute wounds at an early infection stage. Future research is warranted to assess the therapy's effectiveness against more sophisticated infections, especially those involving polymicrobial biofilms. Nonetheless, the most practical and significant application of blue light therapy is on wounds prepared by clinical procedures that disrupt biofilms and remove necrotic tissues, optimizing wound beds for healing.^[^ ^13^, ^57^, ^58^ ^]^ Such procedures are routine particularly in the management of chronic wounds and in preparing wound beds for skin grafting.^[^ ^59^, ^60^ ^]^ With biofilms/necrotic tissues disrupted and dispersed on the wound surface, it creates a window to employ blue light therapy for wound infection control. While our study demonstrated the feasibility of continuous light exposure for 2 days, extending the treatment from several days up to several weeks could be achievable without significant phototoxicity. Investigating the impact of various intermittent illumination patterns, such as 16‐h on followed by 8‐h off, could also prove beneficial to accommodate the diverse needs and circumstances of patients.

The utilization of low irradiance levels, in the range of 2–10 mW/cm^2^, significantly aids the development of photonic wound patches. Building on the antimicrobial property, other functionalities or factors could be integrated toward their specific clinical applications. For example, negative pressure wound therapy^[^ ^57^, ^58^, ^61^, ^62^ ^]^ is a common treatment for chronic wounds and extensive traumas prone to infection. Integrating light‐bathing with negative pressure wound therapy devices could offer sustained infection control and accelerate wound healing. While gallium nitride‐based blue LED technology is suitable for many cases, exploring flexible organic LEDs, fiber‐optic patches, and wireless‐powered light sources^[^ ^63^, ^64^, ^65^ ^]^ opens up innovative possibilities to accommodate various wound shapes, treatment lengths, and patient mobility needs. Additionally, patient factors, e.g., patch breathability, patient comfort/mobility, need to be further considered in the patch design.

## 4 Experimental Section

### Experimental Design

This study aimed to comprehensively evaluate the phototoxicity and effectiveness of the light‐bathing therapy on in vivo rat models of wound infection based on the concept initiated in vitro. For the rat study, randomized controlled laboratory experiments were designed accordingly. For phototoxicity evaluation, each healthy rat was randomly assigned to different groups with an LED array patch attached individually and then treated for approximately 2 days at various irradiance levels for each group. The readouts included visual assessment of erythema/edema formation, endpoint histopathology assessment of phototoxicity, and longitudinal skin temperature monitoring during treatment. The detailed protocol was described in the section: in vivo phototoxicity study on healthy skin. For antimicrobial effectiveness evaluation, rat models of abrasion wound infection were randomly assigned to different groups, each with an LED array patch attached individually, and then treated for approximately 2 days at an irradiance of 5 mW/cm^2^ for the light‐treated group or 0 mW/cm^2^ for the untreated group. The readouts included bacterial bioburden quantified via either longitudinal BLI or endpoint punch biopsy‐based CFUs, visual assessment of wound appearance, bacterial distribution within biopsied wound tissue depicted by FISH imaging, and endpoint histopathology or immunohistochemistry of key cytokine markers within biopsied wound tissue. The detailed protocol was described in the section: in vivo study on abrasion wounds. The sample size for each in vivo rat study is summarized in Table S1 (Supporting Information). The sample sizes for essential experiments used to establish the maximum permissible irradiance and to quantify bacterial bioburden via endpoint punch biopsy‐based CFUs were set at 5 and 7, respectively. These numbers exceed the necessary sample size of 4 determined by power analysis in *R* to detect an estimated 30% difference between the light‐treated and untreated groups with a power of 90% and a two‐sided α of 5%. For CFU‐based bacterial bioburden quantification, a larger sample size was used to compensate for individual rat experiments that failed during the study. Any rat with an incomplete treatment course due to device issues was excluded as specified in Table S1 (Supporting Information). The results of these experiments were confirmed via other independent methods including histopathology, BLI, clinical wound signs, and FISH imaging. For in vivo BLI, the imaging was performed longitudinally at seven consecutive time points for each rat. Three rats were included in each group, which was sufficient to demonstrate the bacterial bioburden trend and reproducibility. Both technical and biological replicates were further specified in each study to ensure reproducibility. The investigators conducting the experiments were not blinded to the interventions applied to each rat.

### Bacterial Strains and Chemicals

This study utilized bacterial strains, including MRSA (USA300), MRSA (USA300 LAC *lux*), *S. aureus* Δ*katA* (sourced from Dr. George Y. Liu's Lab at UCSD), *P. aeruginosa* (PAO1 LAC *lux*), *P. aeruginosa* (CDC AR Bank #0231), *P. aeruginosa* (ATCC 27853), and *E. coli* (ATCC 25922). The chemicals employed included DMTU (D188700, Sigma–Aldrich), BHI medium (BD Difco 237500, Fisher Scientific), mannitol salt agar (1054040500, Sigma–Aldrich), cetrimide agar (OXCM0579B, Thermo Fisher Scientific), poly‐D‐lysine hydrobromide (P0899, Sigma), M9 medium (A1374401, Thermo Fisher Scientific), and Mueller‐Hinton agar (R454082, Thermo Fisher Scientific).

### Bacterial Culture Condition

Bacterial strains were preserved in BHI medium enriched with 20% (v/v) glycerol at ‐80 °C. For experimental use, frozen stocks were thawed and streaked on BHI agar plates, then incubated overnight at 37 °C to facilitate colony development for future use. A stationary‐phase bacterial inoculum was prepared by transferring colonies from previously streaked agar plates into a sterile BHI medium and incubating them in an orbital shaker set at 100 rpm overnight at 37 °C. Prior to experimentation, bacterial cells were pelleted by centrifugation, washed twice with 1× phosphate‐buffered saline (PBS), and resuspended in 1× PBS.

### Light Sources

For the illumination of in vitro samples, as well as for photothermal heating measurements on the rat dorsal skin in vivo, an LED‐based illumination system was utilized. This system comprised a single‐element LED (M405L4, Thorlabs), equipped with an adjustable collimation adapter (SM2F32‐A, Thorlabs) for beam focusing, and powered by a dedicated LED driver (LEDD1B, Thorlabs) connected to a power supply unit (KPS201, Thorlabs). The LED emitted light centered at 409 nm with a 9‐nm bandwidth, capable of producing a maximum output power of 1 W. The output power was adjustable and set to operate in continuous mode, with the beam size tailored via the collimation adapter for precise application. The single‐element LED and the study subjects (in vitro agar plates, well plates, and rats for photothermal heating measurements) were positioned at a fixed distance from each other, maintaining a separation of approximately 30 cm. For in vivo rat studies, a 10×10 LED array (1DGL‐JC‐100W‐405, Chanzon) with a center wavelength of 409 nm, a bandwidth of 11 nm, and a peak output capacity of 100 W was used. This array covered a 22×22 mm^2^ area and provided an emission angle ranging between 120° and 140°, ensuring broad and even coverage. The irradiances and output powers of these lighting systems were measured using a power meter (S121C and PA400, Thorlabs). Additionally, the emission spectra were evaluated with a spectrometer (CCS200, Thorlabs).

### In Vitro Antimicrobial Therapy

In the study of the antimicrobial effects of blue light, experiments on both agar plates and in broth media were conducted. For the study on agar plates, stationary‐phase bacterial inocula were uniformly streaked onto agar plates (e.g., Mueller‐Hinton agar plates). These plates, covered with transparent lids, were then exposed to blue light from an LED device within a 37 °C incubator. The illumination targeted a 1 cm‐diameter area on the plates and was maintained overnight (>10 h) at varying irradiances. Post‐illumination, the formation of bacterial colonies in both illuminated and non‐illuminated zones was assessed visually. For the study in broth medium, stationary‐phase bacterial inocula were introduced into diluted BHI medium (diluted 5× to minimize optical attenuation; refer to Figure S2, Supporting Information), and 30 µL of this bacterial suspension was allocated to each well of a 96‐well plate. The plate was then placed in a 37 °C incubation chamber on the sample stage of a laser‐scanning confocal microscope (FV3000, Olympus), where blue light from multiple single‐element LED sources was directed onto the wells through an auxiliary port of the microscope.

### Temperature Measurements

To assess the thermal impact of blue light exposure, temperature measurements on in vivo rat skin exposed to air using an infrared thermometer (DT8011H, Thermco Products) were conducted. The hair on the rats’ dorsal skin was removed a day prior to the experiment. Under anesthesia, two distinct dorsal skin areas (2×2 cm^2^ each) were illuminated with two separate LED beams set at 5 and 10 mW/cm^2^, respectively, while adjacent, non‐illuminated skin areas served as controls. Additionally, the temperature of the LED arrays without active cooling components during continuous illumination was monitored using the same infrared thermometer.

### Confocal Imaging

For the time‐lapse confocal imaging studies, a setup integrating a single‐element LED with a laser scanning confocal microscope (FV3000, Olympus) was utilized. Bacterial suspensions allocated into a 96‐well plate were positioned within an incubation chamber on the microscope. The LED's collimated beam was precisely aimed at the targeted wells, with special attention to calibrating the irradiance reaching the bacterial suspension in the wells considering the optical attenuation caused by both the incubator window and the plate lid. Time‐lapse images were captured using an UPlanFL N 20× objective (Olympus, NA 0.5) and subsequently processed and analyzed with ImageJ software.

### Animals

All animal experiments conducted in this study received approval from the Institutional Animal Care and Use Committee (IACUC) of Massachusetts General Hospital, ensuring compliance with the National Institutes of Health guidelines (Approval Number: 2022N000039). Male Sprague Dawley rats (CD® Sprague Dawley IGS, 11 weeks old) were sourced from Charles River Laboratories (Massachusetts, USA). The in vivo animal studies and the corresponding animal number of each study are summarized in Table S1 (Supporting Information).

### Wearable Photonic Wound Patch Devices

For the in vivo studies with rats, tethered wearable wound patches incorporating a 10×10 LED array (1DGL‐JC‐100W‐405, Chanzon) were designed. To manage heat, a heat sink (345‐1103‐ND, Digi‐Key) was affixed directly to the LED array's metal backing plate. Additionally, a compact cooling fan (1570‐1113‐ND, Digi‐Key) was secured atop the heat sink using super glue for enhanced thermal management. Electrical wiring connected to the LED and fan was fully encased within a metal spring (PS115H, Instech Laboratories) for protection and flexibility. The proximal end of the spring was secured to the LED headpiece with super glue and metal wires, with its distal end connecting to an electrical slip ring (ZC‐MOFLON‐Slipring, Taida), facilitating rotation without wire tangling. This slip ring, featuring a 12.7‐mm inner hole and a 33‐mm outer diameter, accommodated up to six independent wires, ensuring reliable connection to a power supply (22‐9130C‐ND, Digi‐Key). Customized rat cages were developed to accommodate the rotary slip rings, the wearable light devices, and the rats’ feeding requirements, with the slip ring's stationary side mounted on the rat cage. A four‐pin quick connector streamlined the attachment and detachment process of the wound patch system from the cage. For animal studies, abrasion wounds were initially covered and sealed (non‐breathable) with a transparent film drape (V.A.C. Drape, 3Mm) to prevent external contamination. This drape was not expected to affect wound healing or antimicrobial response (with an optical transparency of 92% at 410 nm) as it was commonly used in negative pressure wound therapy. A customized plastic splint was utilized to stabilize the wound area beneath the drape, ensuring optimal alignment of the LED array with the wound site. The LED device was mounted on the rat by using a custom 3D‐printed frame made from high‐temperature V2 resin (Formlabs), which maintained a 5‐mm gap between the LED output facet and the tissue to avoid direct contact. This frame was securely fastened to the rat dorsum, ensuring stable positioning throughout the study. The quality of each LED array used in the study was similarly assessed by evaluating its brightness at the single‐LED‐element level followed by quantitative evaluation of its irradiance uniformity across the emission facet both right before mounting the LED patch onto each rat and right after removing the LED patch from each rat.

### In Vivo Phototoxicity Study on Healthy Skin

The phototoxicity of the blue light‐bathing therapy was first assessed on the healthy dorsal skin of rats. On the first day, under isoflurane anesthesia, rats had their dorsal and abdominal hair shaved, followed by the application of hair removal cream for complete hair removal. Subsequently, the dorsal skin was cleaned with a 4% chlorhexidine gluconate solution before mounting a transparent film drape (V.A.C. Drape, 3M) and the LED device over the shaved area. Starting on the second day, the LED device was activated to deliver varying irradiances in a stepwise reduction (from 30 to 20, 10, 5, and 0 mW/cm^2^ at the skin surface) over continuous illumination periods ranging from 36 to 48 h. Following a 6‐h illumination period at 30 mW/cm^2^, with one rat showing good tolerance, the full‐course illumination was extended on this rat, followed by another rat at 20 mW/cm^2^. Subsequently, two rats were subjected to 10 mW/cm^2^ illumination, and five rats were assigned to both the 5 mW/cm^2^‐treated and untreated groups. After two‐day exposure (on Day 4), the rats were re‐anesthetized for device removal and initial wound condition documentation through photography. The animals were then returned to their cages for continued skin reaction observation over the next three days, culminating on Day 7. Final photographs were taken before the rats were euthanized for tissue biopsy collection. These samples were subjected to H&E and TUNEL staining for comprehensive histopathological analysis of the skin's response to the light exposure.

### In Vivo Study on Abrasion Wounds

The impact of phototoxicity and the therapeutic efficacy of the light‐bathing therapy was assessed on rat dorsal skin subjected to abrasion wounds with or without induction of bacterial infection. Following the initial hair removal procedure, identical to the healthy skin phototoxicity study, on the subsequent day, rats were anesthetized with isoflurane and received buprenorphine intraperitoneally for analgesia. Using a scalpel, two similar abrasion wounds (15×15 mm^2^ each), maintaining a separation of approximately 20 mm along the dorsal spine, was meticulously created by repeatedly scratching the skin to affect the stratum corneum and upper layer of the epidermis without damaging the dermis. Minimal to no bleeding was observed post‐wounding. Custom plastic splints were applied around each wound to ensure a flat surface for consistent healing and light exposure. Each wound was then covered with a film drape that wrapped around the rat's body, with an LED device positioned over the upper wound for 5 mW/cm^2^ light‐bathing treatment. The placement near the head discouraged interference with the device's wiring. The lower wound served as a control, receiving identical preparation without light‐bathing treatment. After approximately 48 h, the animals were euthanized, and the devices and drapes were removed for follow‐up analysis. This phase of the study utilized two rats, yielding four wound sites for examination. For the investigation into the effects of light‐bathing therapy on wounds with acute bacterial infection, overnight cultures of MRSA USA300 *lux*, MRSA USA300, *P. aeruginosa* PAO1 *lux*, or *P. aeruginosa* CDC AR Bank #0231 were prepared, washed in 1× PBS, and adjusted to a density conducive to a bacterial inoculation load of approximately 10^8^ CFUs. A 20‐µL aliquot of the bacterial suspension was spread evenly across each wound surface. After allowing the inoculum to dry, wounds were sealed with film drapes, and the LED device was affixed for subsequent light treatment. Rats were then housed in customized cages to allow for the development of acute infection over 3 h. Following this infection period, light therapy commenced. Three rats for each bioluminescent strain and seven rats for each non‐bioluminescent clinical strain were used (one rat in the *P. aeruginosa* CDC AR Bank #0231‐infected group and two rats in the MRSA USA300‐infected group were excluded due to device damage during light treatment), and three to five 3‐mm punch biopsies were randomly sampled from each wound for subsequent tissue CFU enumeration, PNA‐FISH imaging, or histopathological examination to assess the effects of the light treatment on wound healing and infection control.

### Bioluminescence Imaging

The in vivo BLI was executed using the IVIS Lumina In Vivo Imaging System (PerkinElmer). Initial tests on in vitro agar plates established the BLI signal's linearity and minimum detection sensitivity for the bioluminescent strains. Considering the bioluminescence intensity variance between MRSA USA300 *lux* and *P. aeruginosa* PAO1 *lux*, the integration times were set at 240 and 24 s, respectively, for optimal signal‐to‐noise ratio while without signal saturation. Bioluminescent strains were used to prepare rat models of abrasion wound infection, with three rats per group. BLI was conducted at various intervals up to 44 h. For BLI, rats were anesthetized and then positioned in the IVIS system equipped with anesthesia. The imaging process, including device removal and reattachment, was efficiently completed within 10 min. After imaging at the final time point, rats were euthanized, and wound conditions were documented. BLI data were processed and analyzed with ImageJ software, providing critical insights into the dynamic bacterial presence within the wounds.

### CFU Enumeration Assay

To assess the viable bacterial load within each wound, three 3‐mm punch biopsies were randomly sampled from each wound followed by a tissue CFU enumeration assay. The biopsy samples were collected into tissue lysing tubes (Lysing Matrix D, 2 mL tube, MP Biomedical) containing 800 µL of 1× PBS. They were then lysed by using a high‐speed tissue homogenizer (FastPrep‐24 Classic Instrument, MP Biomedical) for thorough tissue disruption. Subsequently, 200 µL of the lysate was transferred to a 96‐well plate for 10‐fold serial dilutions to quantify CFUs for each sample. Mannitol salt agar (Sigma–Aldrich) and cetrimide agar (Thermo Fisher Scientific) plates were utilized for selective isolation of *S. aureus* and *P. aeruginosa*, respectively. Plates were incubated at 37 °C until visible colonies formed for CFU enumeration. To enhance detection sensitivity, an additional 100 µL of homogenate underwent glass‐bead plating across the entire surface of the agar plate, amplifying the detection sensitivity by 25‐fold. Each CFU measurement included three technical replicates. The initial bacterial inoculation load in each rat experiment was similarly quantified. These measurements provided a comprehensive analysis of bacterial load both pre‐ and post‐treatment.

### PNA‐FISH Imaging

Following protocols from prior studies,^[^ ^66^, ^67^ ^]^ Alexa488‐tagged and Cy5‐tagged, 16S rRNA PNA probes developed in a PNA format by PNA Bio Inc were used to specifically target *S. aureus* and *P. aeruginosa*, respectively. A probe validation test was conducted first. Cultured MRSA USA300 or *P. aeruginosa* CDC AR Bank #0231 were fixed in 10% formalin for 1 h and subsequently stored in 50% (v/v) ethanol at ‐20 °C. Fixed cells were pelleted, rinsed, and resuspended in a hybridization solution containing dextran sulfate (10% wt/vol), NaCl (10 mMm), formamide (30% v/v), sodium pyrophosphate (0.1% wt/vol), polyvinylpyrrolidone (0.2% wt/vol), Ficoll (0.2% wt/vol), disodium EDTA (5 mMm), Triton X‐100 (0.1% vol/vol), Tris‐HCl (50 mMm, pH 7.5), and the respective PNA probe (200 nMm). This mixture was incubated at 57 °C for 2 h for hybridization. Afterward, cells were centrifuged at 17000 g for 6 min, resuspended in 500 µL of wash solution containing Tris Base (5 mMm), NaCl (15 mMm), and Triton X (1% vol/vol, pH 10), incubated at 57 °C for 90 min, and then pelleted and resuspended in 1 mL of sterile water. The prepared cells were placed on a glass slide, air‐dried, and covered with ProLong Gold antifade mounting medium (Thermo Fisher Scientific, P36930) between the glass substrate and cover glass (VWR international, 48368‐040). Imaging was performed with a Zeiss LSM780 confocal laser scanning microscope using a 63× oil immersion objective (NA 1.4) to capture Alexa488 and Cy5 fluorescence in a sequential‐scanning mode with a field of view of 135 µm×135 µm. With the successful probe validation, PNA‐FISH imaging for rat wound sections was further performed following a similar protocol but with hybridization conducted on UltraFrost adhesion slides (VWR international, 50‐301‐70). Paraffin‐embedded punch biopsy sections from control and 5mW/cm^2^‐treated wounds were placed on top of the UltraFrost adhesion slides followed by hybridization inside a temperature‐controlled oven (57 °C for 120 min). Post‐hybridization with the hybridization buffer removed, wound sections were washed for 90 min at 57 °C, air‐dried, and further covered with ProLong Gold antifade mounting medium between the glass substrate and cover glass. Imaging was performed by utilizing the confocal microscope (Zeiss LSM780) with a UPlanFL N 20× objective (Olympus, NA 0.8), with a field of view of 425 µm×425 µm, to visualize the fluorescently tagged bacterial cells within the wound sections.

### Histopathology and Immunohistopathology

Histological and immunohistochemical analysis, including H&E, TUNEL, and immunohistochemistry, were conducted on punch biopsy samples for this study. Immediately after collection, these samples were fixed in 4% paraformaldehyde to preserve tissue architecture and cellular details. Following adequate fixation, the specimens were embedded in paraffin wax, and thin sections of 5 µm thickness were prepared and mounted on cover glasses for staining. The TUNEL staining, utilizing DeadEnd^TM^ Fluorometric TUNEL System from Promega, was employed to identify apoptotic cells within the tissue by highlighting fragmented DNA. For the immunohistochemical staining, three pro‐inflammatory cytokines was targeted: TNF‐α, IL‐6, and IL‐1β. This was achieved using specific rabbit polyclonal antibodies: anti‐TNF‐α (ab6671, Abcam), anti‐IL‐6 (ab229381, Abcam), and anti‐IL‐1β (ab9722, Abcam). Following primary antibody incubation, sections were treated with a compatible secondary antibody (RALP525, Biocare Medical) and visualized using Warp Red Chromogen kit (WR806, Biocare Medical) to detect the antigens of interest. Finally, all stained slides, including those processed for H&E and TUNEL, were digitized using a NanoZoomer slide scanner (Hamamatsu) for detailed image analysis and documentation of histopathological changes.

### Statistical Analysis

The number of biological and technical replicates was indicated in the corresponding figure legends. Data in this study were presented as mean ± standard deviation for analyzing technical replicates and mean ± standard error for analyzing biological replicates. Unless specified otherwise, data were presented as mean ± standard deviation. Comparisons between the two datasets were made using an unpaired two‐tailed Student's *t*‐test. A *P* value < 0.05 was deemed to indicate a statistically significant difference, while *P* < 0.01 and *P* < 0.001 were considered indicative of even more significant differences. The notation “ns” denotes a lack of significant difference.

## Conflict of Interest

The authors declare no conflict of interest.

## Author Contributions

J.H. and W.M. contributed equally to this work. J.H. and S.H.Y. designed the project. J.H. handled in vitro experiments, LED device development, and animal protocol creation. J.H. and W.M. performed rat studies. W.M. interpreted histology. P.T.D. performed FISH imaging. C.D.A. and H.Y. assisted in animal studies. L.N. helped with in vitro experiments. Y.W. and J.T. contributed to animal protocol. T.D. contributed to lab resources. J.G. provided clinical insights, R.R.A., J.T., J.A.G., and S.H.Y. conceived this project and secured funding. J.H., W.M., and S.H.Y. performed data analysis, prepared figures, and wrote the manuscript with input from all co‐authors.

## Ethical Statement

All animal experiments conducted in this study received approval from the Institutional Animal Care and Use Committee (IACUC) of Massachusetts General Hospital, ensuring compliance with the National Institutes of Health guidelines (Approval Number: 2022N000039).

## Supporting information



Supporting Information

Supplemental Movie 1

Supplemental Movie 2

Supplemental Movie 3

Supplemental Movie 4

## Data Availability

All data are available in the main text or the supplementary materials. The raw data and materials underlying the graphs presented in this paper are accessible and will be provided upon a reasonable request to the corresponding authors.
