# Supplementary material for: Low‐Irradiance Antimicrobial Blue Light‐Bathing Therapy for Wound Infection Control
Source: Adv Sci (Weinh). 2025 Apr 14;12(20):2412493. doi: 10.1002/advs.202412493 (PMC12120825; doi:10.1002/advs.202412493)
Supplement: Supplementary file 1 — Supporting Information [file ADVS-12-2412493-s001.docx]

Supporting Information

Low-Irradiance Antimicrobial Blue Light-Bathing Therapy for Wound Infection Control

Jie Hui*, Wonjoon Moon, Pu-Ting Dong, Carolina dos Anjos, Laisa Negri, Hao Yan, Ying Wang, Joshua Tam, Tianhong Dai, R. Rox Anderson, Jeremy Goverman, Jeffrey A. Gelfand, and Seok-Hyun Yun*

This Supplementary Information file includes:

Figure S1 to S16

Table S1

Legend for Movie S1 to S4

Other Supplementary Materials for this manuscript include the following:

Movie S1 to S4

**
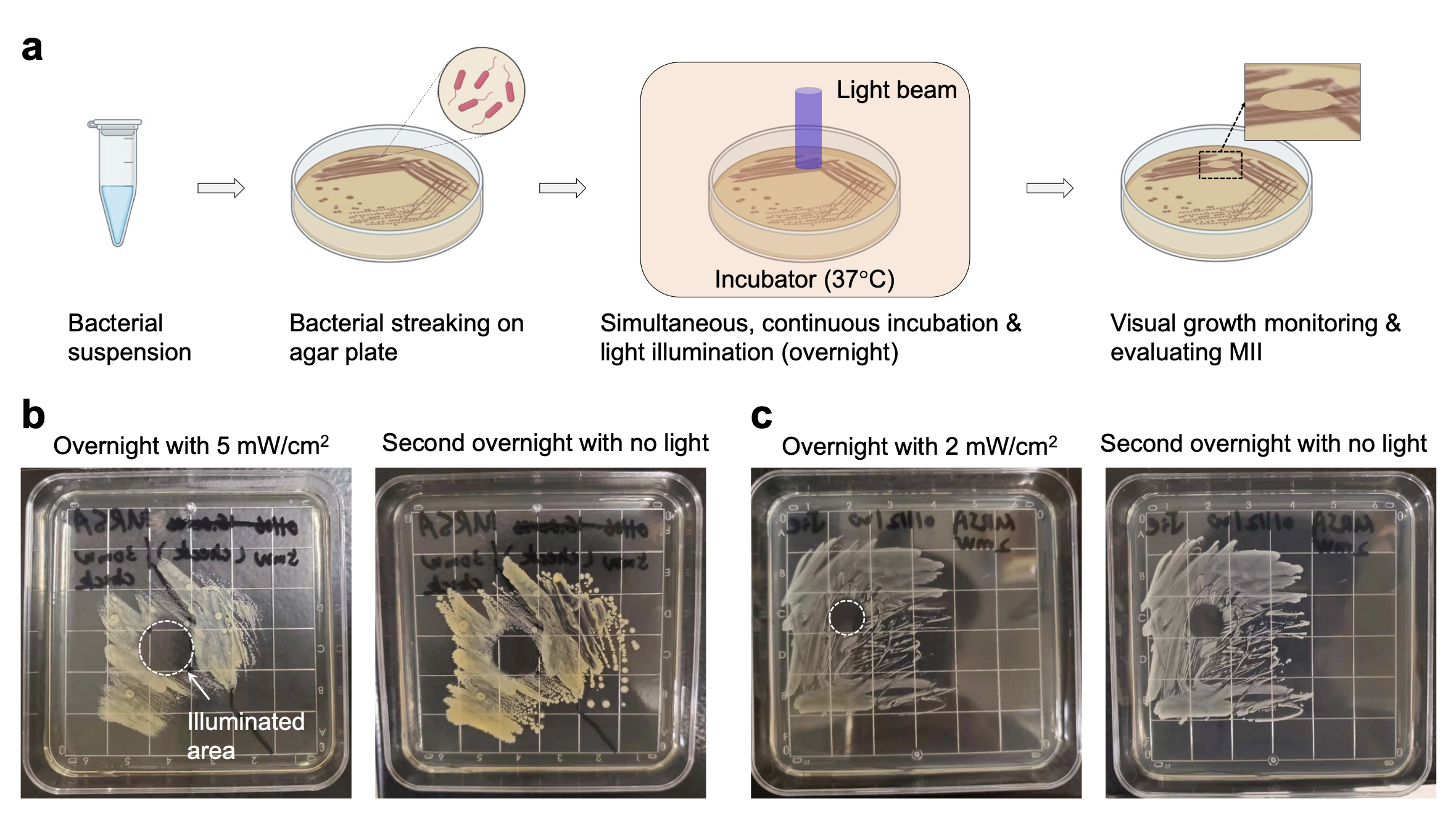
**

**Figure S1.** Quantification of minimum inhibitory irradiance (MII) using *in vitro* streaked agar plates. (a) Schematic of the measurement protocol. (b) Bacterial colony formation on an agar plate after overnight incubation under 5 mW/cm^2^ light (left), followed by another overnight incubation without light illumination (right). (c) Another agar plate showing bacterial colony formation after overnight incubation under 2 mW/cm^2^ light (left), followed by another overnight incubation without light illumination (right). The plate grid size is 1 cm. The bacteria in the illuminated areas (dashed circles) are inhibited or eradicated while bacteria outside the illumination zones continue to proliferate.


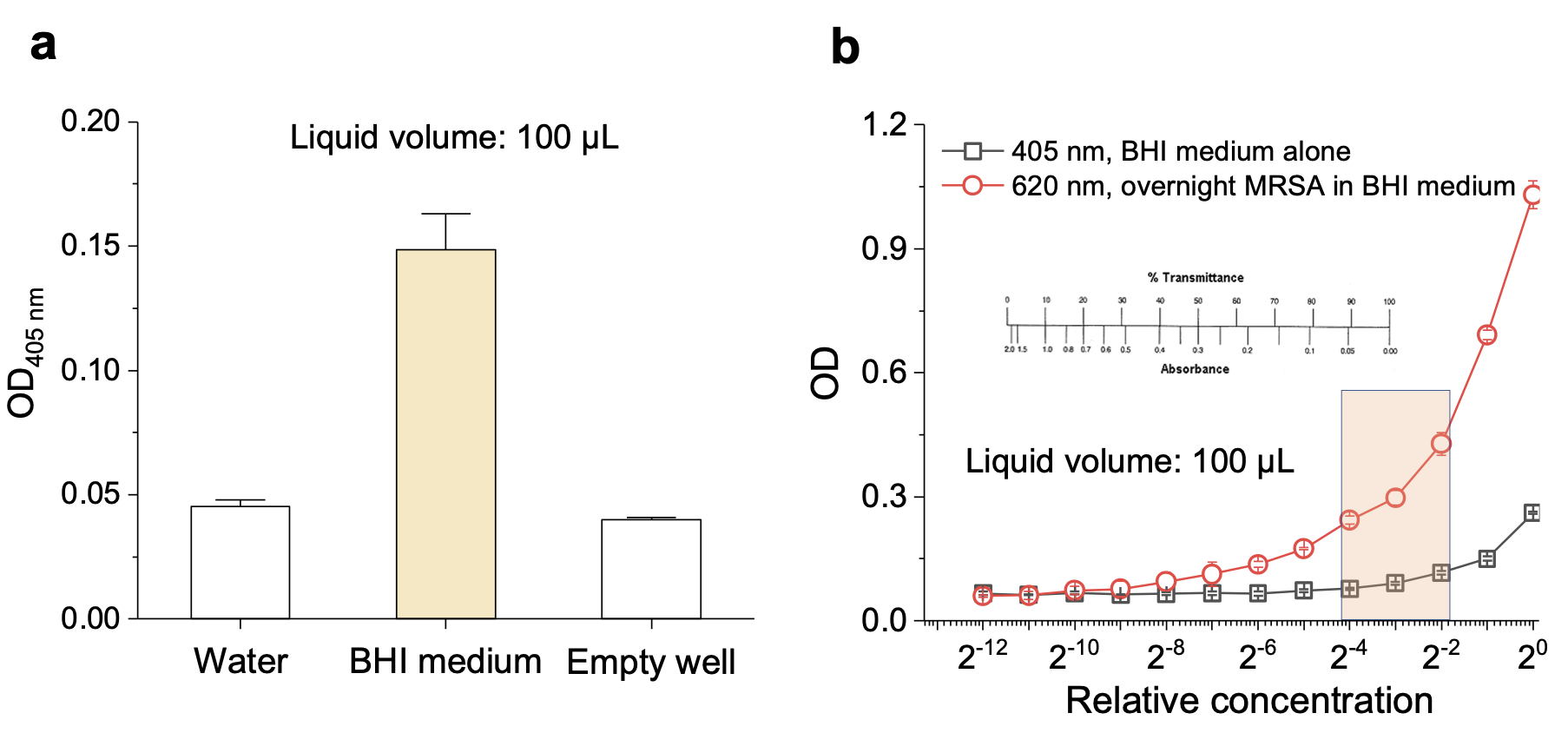


**Figure S2.** Light attenuation at 405 nm by nutrient-rich brain heart infusion (BHI) medium. (a) Optical density (OD) at 405 nm measured from an empty well, 100 μL water in a well, 100 μL BHI medium in a 96-well plate. (b) OD measurement at 405 nm of pure BHI medium with different dilution factors, along with OD at 620 nm of overnight-incubated MRSA USA300-containing BHI medium.


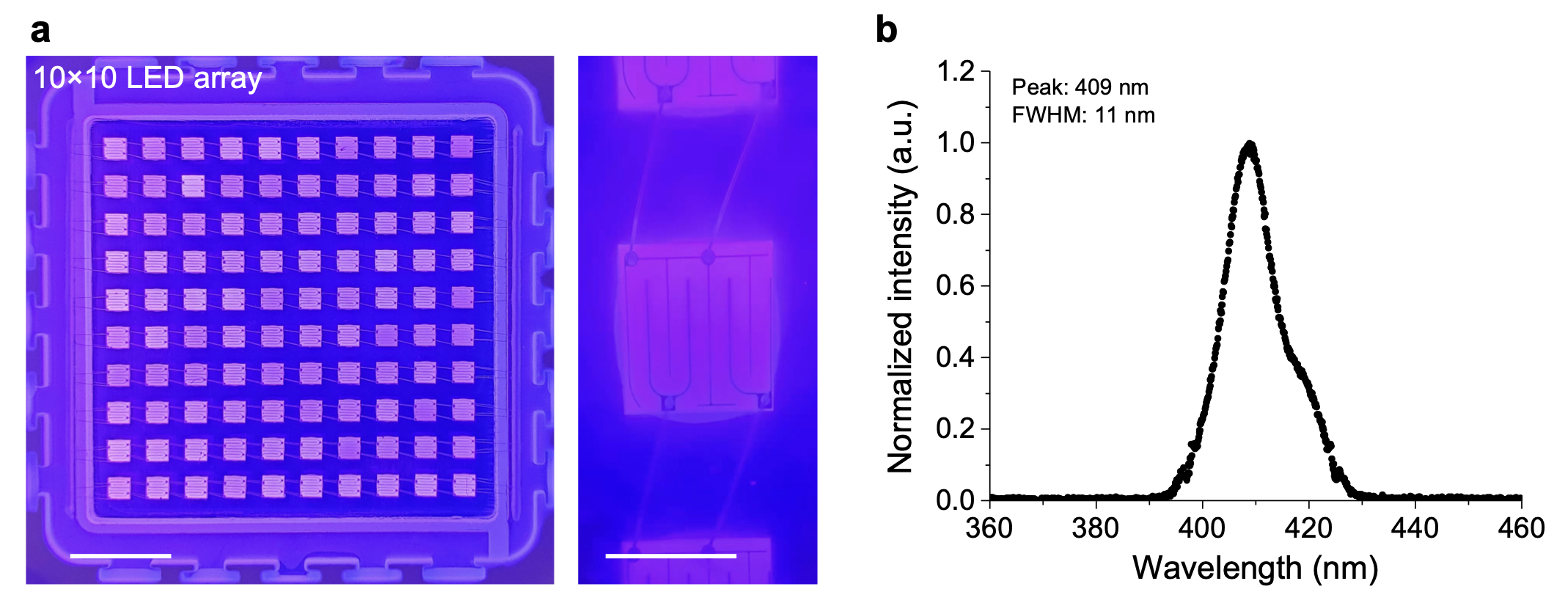


**Figure S3.** Emission characteristics of a typical LED array. (a) (Left) Photograph of an LED array under active emission (1DGL-JC-100W-405, Chanzon). Scale bar, 4 mm. (Right) A zoom-in image of a single LED element, with a scale bar of 1 mm. These images show a qualitative assessment of irradiance uniformity from element to element. (b) Emission spectrum of the LED array.


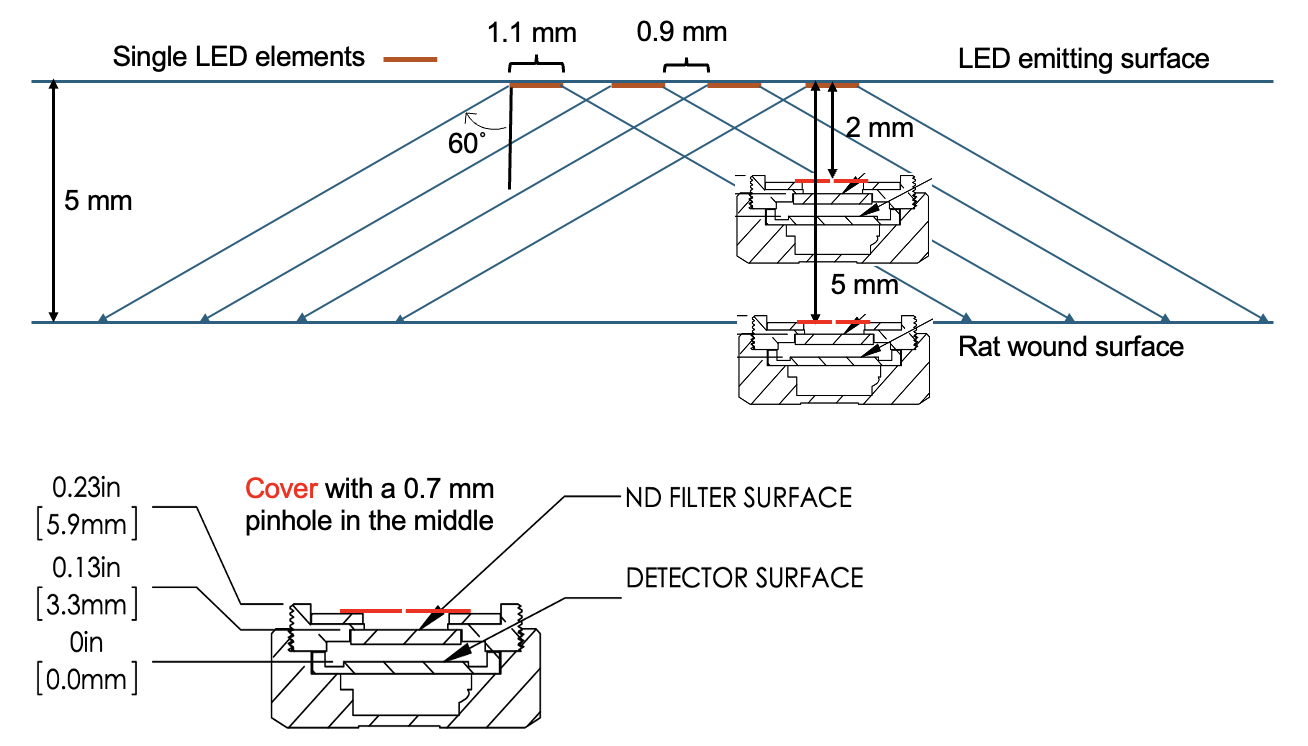


**Figure S4.** Schematics of LED array’s geometrical specifications, its irradiance uniformity characterization geometry, and the structure of the powermeter sensor head. In our irradiance uniformity characterization for each LED array, irradiance profiles over skin-wound-equivalent areas were measured using a power meter (S121C and PA400, Thorlabs). The photodiode power sensor head was placed right beneath the LED array resulting in a 5.9-mm distance from LED array emission facet to photodiode surface. The sensor head was then scanned across the area (in the X-Y plane) to assess the uniformity. This configuration closely reproduced our *in vivo* illumination geometry, where a mounting frame was employed to maintain a distance of approximately 5 mm from the LED array emission facet to the skin wound. Throughout our study, we ensured that the measured irradiance values had a coefficient of variation less than 10%. To obtain the precise irradiance distribution map, we covered the sensor head with black tape (100% blockage) but with a small 0.7-mm-diameter hole generated in the middle, and then scanned the sensor head within the 15×15 mm^2^ skin-wound-equivalent area with a step size of 1 mm at depths of 2 mm and 5 mm (from the LED array emission facet to the black tape), respectively.


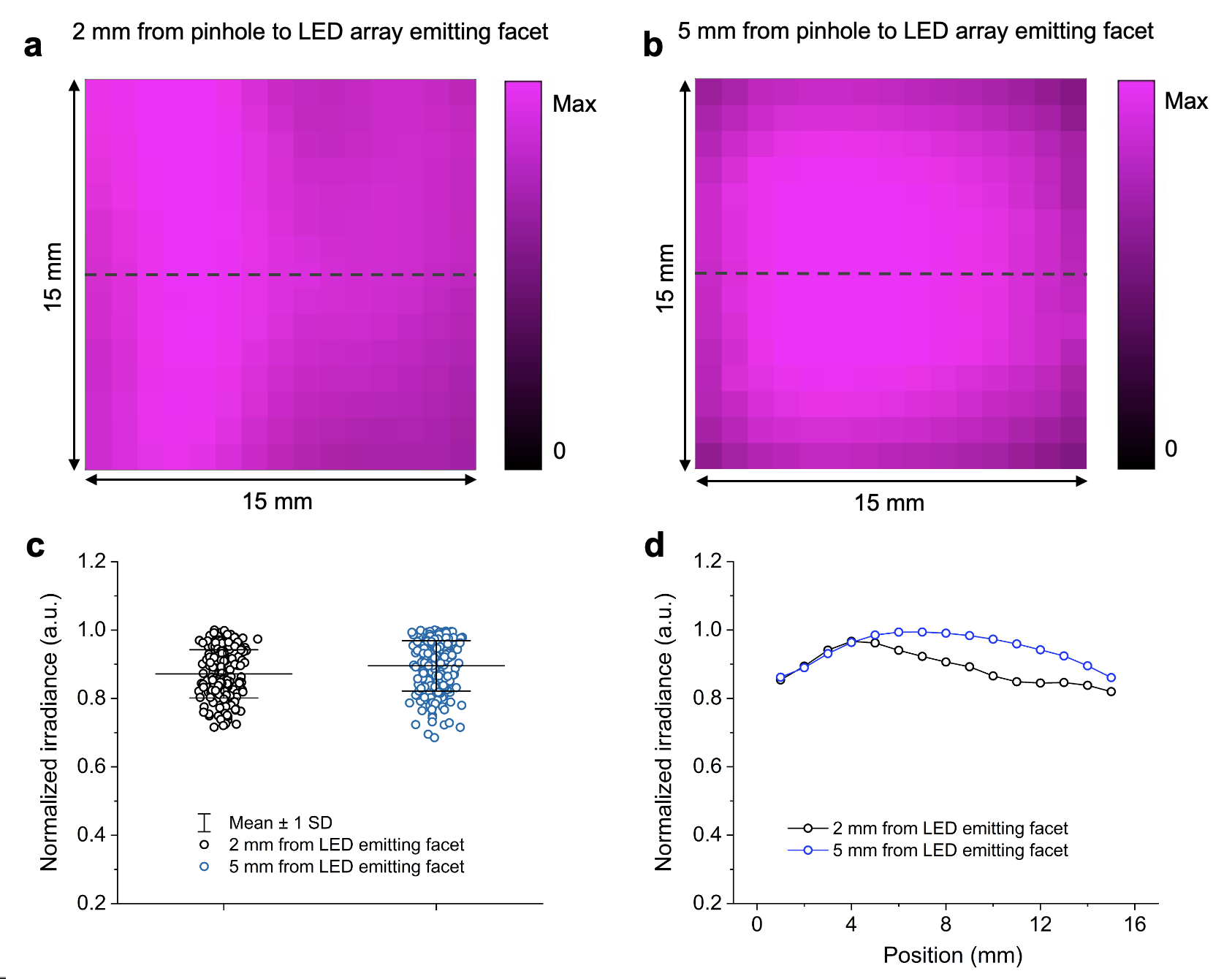


**Figure S5.** Irradiance uniformity of a typical LED array characterized at different depths via a small pinhole-based mapping method, as shown in Figure S4. Normalized irradiance distribution map at 2 mm (a) and 5 mm (b) away from the LED array emission facet. (c) Plot of the normalized irradiance values from (a) and (b) across the entire 15×15 mm^2^ area with coefficient of variation of approximately 8% as calculated for each. (d) Plot of the normalized irradiance values across the dashed lines in (a) and (b). Pinhole size, 0.7 mm in diameter.

**Figure S6.** Design and images of the LED device mounting frame, showing the top view (left) and bottom view (right). The mounting frame was 3D-printed using High Temp V2 resin and a 3D printer (both from Formlabs). Its height was designed to maintain an approximately 5 mm distance between the LED emission facet and skin/wound surface. Its positioning tab was used to firmly attach the mounting frame, along with the LED device, onto rat dorsum using strips of adhesive film drape.


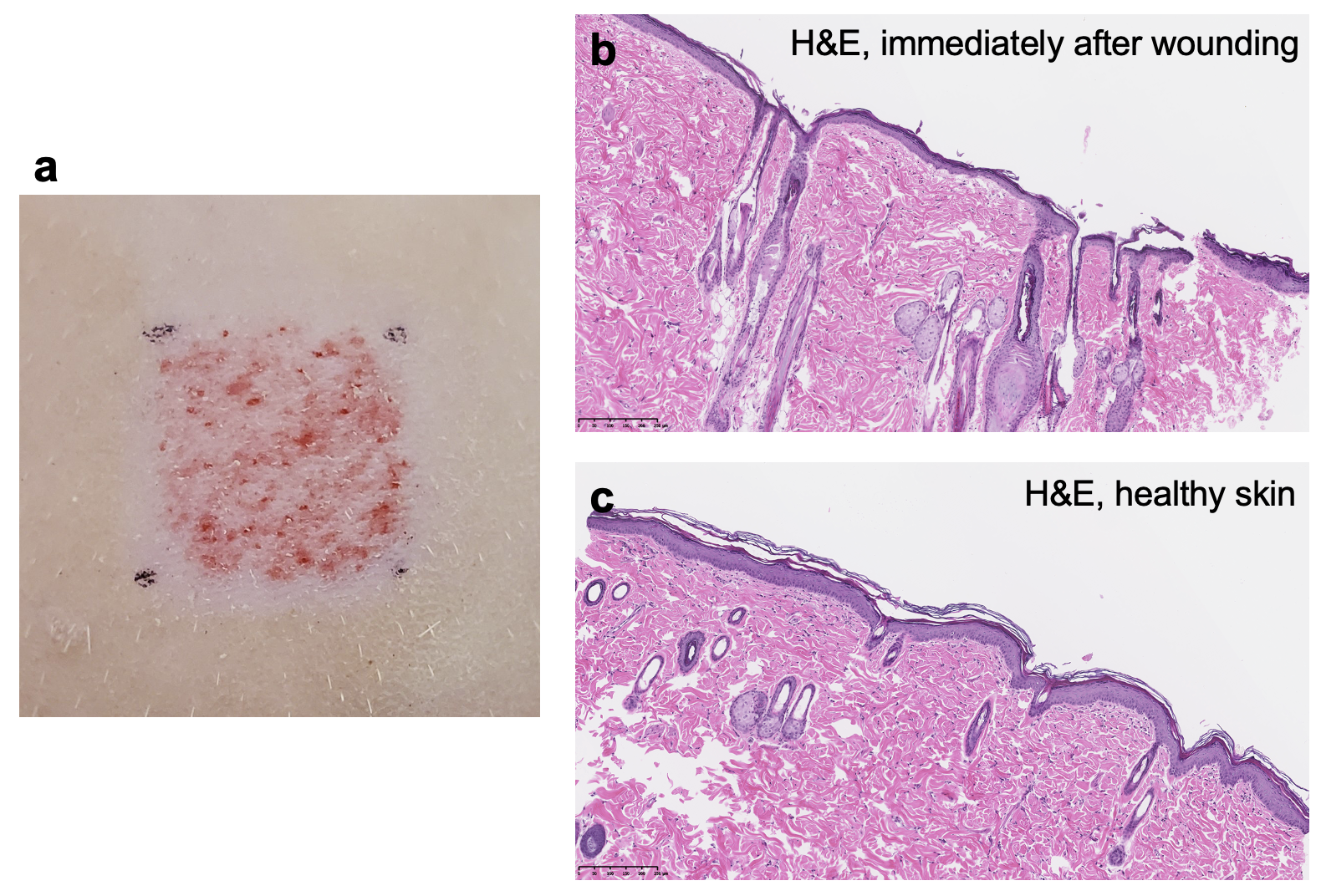


**Figure S7.** Characterization of abrasion wound induced on rats. (a) Photo of a freshly induced abrasion wound on rat dorsal skin with an area of 15×15 mm^2^. Representative H&E histology image of the abrasion wound (b) and healthy skin (c). Given the level of abrasion shown in (a), more significant disruption of the epidermis layer within the scattered spots showing minor bleeding was expected. However, in our histology results in (b), we only found minor disruption, which was likely due to the sparse distribution of the small bleeding spots and our small punch biopsy size (3 mm). Scale bar, 250 μm.

**Figure S8.** Measurement showing the linear relationship between bioluminescence (BLI) signal and signal integration time for MRSA USA300 *lux* cells. (a-c) Images of MRSA USA300 *lux* cells inoculated on an agar plate taken at different integration times. The inoculation load was 6.3×10^7^ CFUs over an area of 15×15 mm^2^ (yellow dashed frame in (c)) simulating the bacterial inoculation on a rat wound. Scale bar, 5 mm. (d) BLI signal plot for (a-c) along a diagonal axis (white dashed line in (c)). The detection limit was 14 CFUs under these imaging settings. (e) The integrated BLI signal intensity showing the total BLI bioburden for (a-c) over the integration time.

**Figure S9.** Measurement confirming the linearity between bioluminescence (BLI) signal and bacterial load. (a-e) BLI images of *P. aeruginosa* PAO1 *lux* cells inoculated on agar plates with different dilution factors. The starting inoculation load in (a) was 1.7×10^7^ CFUs, which was uniformly smeared within an area of 15×15 mm^2^ (yellow dashed frame in (a)) simulating the bacterial inoculation on a rat wound. Scale bar, 5 mm. The BLI reached its detection limit at a dilution factor of 10000× in (e). (f) Photo of the *in vitro* agar plate in (a) and a merge with its BLI image. (g) BLI signal plot for (a-e) along a diagonal axis (white dashed line in (a)). Inset, a zoom-in plot. The detection limit was 14 CFUs for *P. aeruginosa* PAO1 *lux* under the imaging settings. (h) The integrated BLI signal intensity showing the total BLI bioburden for (a-e) and having a linear relationship with the bacterial load.

**Figure S10.** Measurement confirming the linear relationship between bioluminescence (BLI) signal and camera integration time for *P. aeruginosa* PAO1 *lux* cells. (a-e) BLI images of bacterial cells inoculated on an agar plate under different integration times. The inoculation load was 1.7×10^7^ CFUs, which was uniformly smeared within an area of 15×15 mm^2^ (yellow dashed frame in (e)) simulating the bacterial inoculation on a rat wound. Scale bar, 5 mm. (f) BLI signal plot for (a-e) along a diagonal axis (white dashed line in (e)). (g) The integrated BLI signal intensity showing the total BLI bioburden for (a-e) over the camera exposure time or integration time.

Control (no light, 2 days)

Light bathing (5 mW/cm^2^, 2 days)

**Figure S11.** Time-lapse bioluminescence imaging (BLI) data of the untreated (top) and treated (bottom) MRSA USA300 *lux*-infected abrasion wounds on two other rats. (Left) Second set of independent replicates. (Right) Third set of independent replicates. The first image in each set shows the photo of the infected wound. Scale bar, 5 mm.


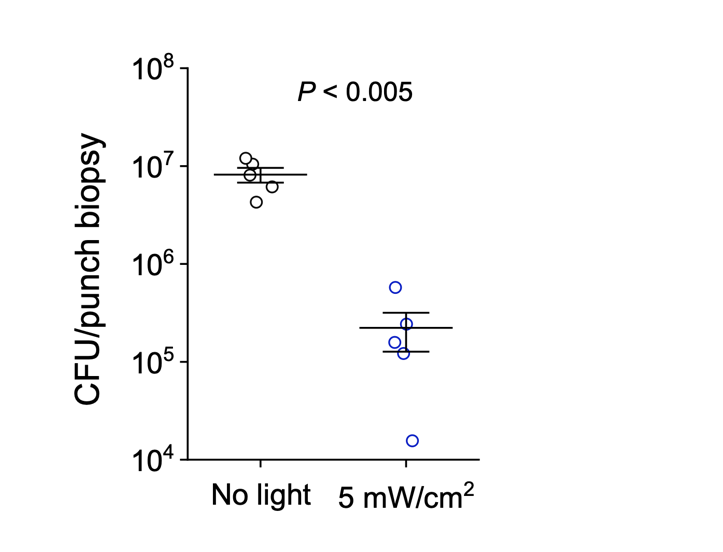


**Figure S12.** CFU per punch biopsy for the untreated or 5 mW/cm^2^-treated groups infected with MRSA (USA300) (from **Figure 4e**). Each CFU value (circle) represents the average of three punch biopsies from each rat. Bars represent mean ± standard error of the group.

**Figure S13.** PNA-FISH imaging of planktonic *S. aureus* (MRSA USA300) and *P. aeruginosa* (CDC AR Bank #0231) for probe specificity validation. No cross hybridization was found between the two FISH probes. Scale bar, 30 μm.


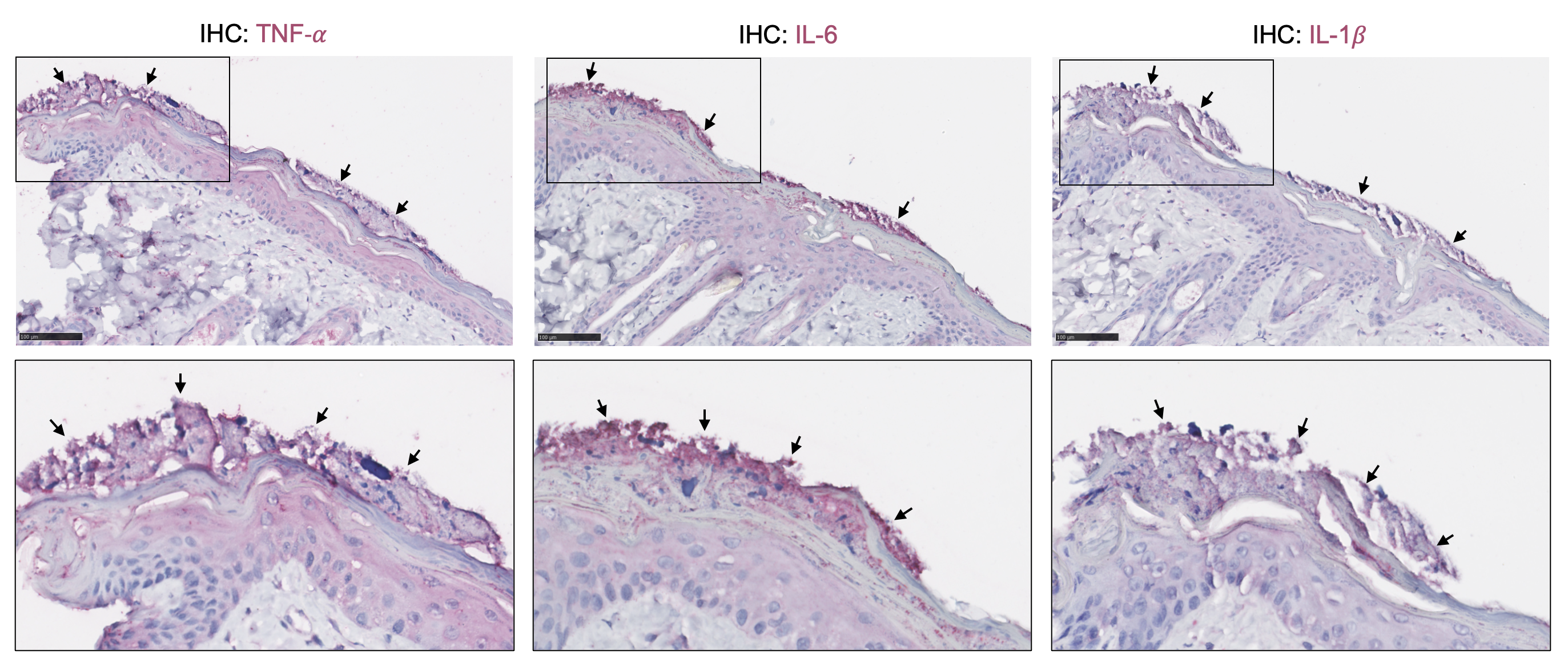


**Figure S14.** Another representative data set for endpoint immunohistochemical staining of several key cytokines for tissue samples collected from the untreated group on *in vivo* rat wound model, which showed more significant epidermis disruption induced by MRSA infection. Arrows indicate the extensive expression of corresponding cytokine markers. Scale bar, 100 μm.

Control (no light, 2 days)

Light bathing (5 mW/cm^2^, 2 days)

**Figure S15.** Time-lapse bioluminescence imaging (BLI) data of the untreated (top) and treated (bottom) *P. aeruginosa* PAO1 *lux*-infected abrasion wounds on two other rats. (Left) Second set of independent replicates. (Right) Third set of independent replicates. The first image in each set shows the photo of the infected wound. Scale bar, 5 mm.

**
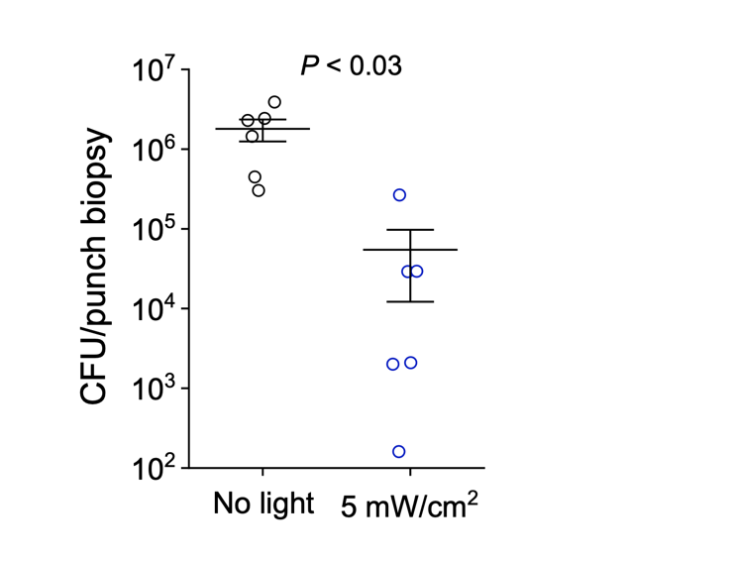
**

**Figure S16.** CFU per punch biopsy for the untreated and 5 mW/cm^2^-treated groups infected with *P. aeruginosa* (CDC AR Bank #0231) (from Figure 5e). Each CFU value (circle) represents the average of three punch biopsies from each rat. Bars represent mean ± standard error of the group.

| *In vivo* rat assays | Inoculated bacterial species/strain | Animal number/wound number | Additional note |
| --- | --- | --- | --- |
| Assay 1: Phototoxicity evaluation on healthy skin | No inoculation | 14 rats with one wound on each (1 rat for 30 mW/cm^2^; 1 for 20 mW/cm^2^; 2 for 10 mW/cm^2^; 5 for 5 mW/cm^2^; 5 for 0 mW/cm^2^) |  |
| Assay 2: Phototoxicity evaluation on abrasion wound | No inoculation | 2 rats with two wounds on each (one wound for 5 mW/cm^2^ bathing therapy; the other serves as control) |  |
| Assay 3: Illumination-induced skin temperature change | No inoculation | 1 rat with three skin areas (one area for 5 mW/cm^2^ illumination; one area for 10 mW/cm^2^; and one area serves as control) |  |
| Assay 4: Bioluminescent *S. aureus*-based IVIS imaging | MRSA (USA300 LAC::*lux*) | 3 rats with two wounds on each (one wound for 5 mW/cm^2^ bathing therapy; the other serves as control) |  |
| Assay 5: Bioluminescent *P. aeruginosa*-based IVIS imaging | *P. aeruginosa* (PAO1 LAC::*lux*) | 3 rats with two wounds on each (one wound for 5 mW/cm^2^ bathing therapy; the other serves as control) |  |
| Assay 6: *In vivo* study on MRSA-infected abrasion wounds | MRSA (USA300) | 7 rats with two wounds on each (one wound for 5 mW/cm^2^ bathing therapy; the other serves as control) | 2 rats were dropped out due to device damage that occurred during light treatment |
| Assay 7: *In vivo* study on *P. aeruginosa*-infected abrasion wounds | *P. aeruginosa* (CDC AR Bank #0231) | 7 rats with two wounds on each (one wound for 5 mW/cm^2^ bathing therapy; the other serves as control) | 1 rat was dropped out due to device damage that occurred during light treatment |
| Assay 8: *S. aureus*-specific FISH imaging | MRSA (USA300) | 3 rats with two wounds on each (one wound for 5 mW/cm^2^ bathing therapy; the other serves as control) | The rats were randomly selected from Assay 6. |
| Assay 9: *P. aeruginosa*-specific FISH imaging | *P. aeruginosa* (CDC AR Bank #0231) | 3 rats with two wounds on each (one wound for 5 mW/cm^2^ bathing therapy; the other serves as control) | The rats were randomly selected from Assay 7. |

**Table S1.** Summary of *in vivo* rat studies and number of rats involved in each assay or study.

**Movie S1.** Time-lapse confocal images of MRSA USA300 cells *in vitro* with concurrent, continuous blue light illumination at different irradiances. Scale bar, 20 μm.

**Movie S2.** Time-lapse confocal images of MRSA USA300 cells *in vitro* supplemented with 16 mM DMTU and further with concurrent, continuous blue light illumination at different irradiances. Scale bar, 20 μm.

**Movie S3.** Time-lapse confocal images of catalase-deficient mutant *S. aureus* Δ*katA* cells *in vitro* with concurrent, continuous blue light illumination at different irradiances. Scale bar, 20 μm.

**Movie S4.** Video of rat models used in the study with and without a light-delivering wound patch.
